# Study of Lysozyme-Loaded Poly-L-Lactide (PLLA) Porous Microparticles in a Compressed CO_2_ Antisolvent Process

**DOI:** 10.3390/ma6083571

**Published:** 2013-08-19

**Authors:** Yong-Qiang Kang, Chen Zhao, Ai-Zheng Chen, Shi-Bin Wang, Yuan-Gang Liu, Wen-Guo Wu, Xiao-Qian Su

**Affiliations:** 1 College of Chemical Engineering, Huaqiao University, Xiamen 361021, China; E-Mails: hqukyq@126.com (Y.-Q.K.); rita88130@163.com (C.Z.); sbwang@hqu.edu.cn (S.-B.W.); ygliu@hqu.edu.cn (Y.-G.L.); wuwenguo@hqu.edu.cn (W.-G.W.); xqsu2013@163.com (X.-Q.S.); 2 Institute of Pharmaceutical Engineering, Institute of Biomaterials and Tissue Engineering, Huaqiao University, Xiamen 361021, China

**Keywords:** PLLA, porous microparticles, PCA process, emulsion, pulmonary drug delivery

## Abstract

Lysozyme (LSZ)-loaded poly-L-lactide (PLLA) porous microparticles (PMs) were successfully prepared by a compressed CO_2_ antisolvent process in combination with a water-in-oil emulsion process using LSZ as a drug model and ammonium bicarbonate as a porogen. The effects of different drug loads (5.0%, 7.5% and 10.0%) on the surface morphology, particle size, porosity, tapped density and drug release profile of the harvested PMs were investigated. The results show that an increase in the amount of LSZ added led to an increase in drug load (DL) but a decrease in encapsulation efficiency. The resulting LSZ-loaded PLLA PMs (LSZ-PLLA PMs) exhibited a porous and uneven morphology, with a density less than 0.1 g·cm^−3^, a geometric mean diameter of 16.9–18.8 μm, an aerodynamic diameter less than 2.8 μm, a fine particle fraction (FPF) of 59.2%–66.8%, and a porosity of 78.2%–86.3%. According to the results of differential scanning calorimetry, the addition of LSZ improved the thermal stability of PLLA. The Fourier transform infrared spectroscopy analysis and circular dichroism spectroscopy measurement reveal that no significant changes occurred in the molecular structures of LSZ during the fabrication process, which was further confirmed by the evaluation of enzyme activity of LSZ. It is demonstrated that the emulsion-combined precipitation with compressed antisolvent (PCA) process could be a promising technology to develop biomacromolecular drug-loaded inhalable carrier for pulmonary drug delivery.

## 1. Introduction

With advantages such as incredible selectivity and the ability to provide effective and potent action, proteins and other biomacromolecular drugs have been widely used in the treatment of numerous diseases, especially in pulmonary diseases [1,2]. However, several challenges, such as a relatively short half-life within the body and low oral and transdermal bioavailabilities are still encountered. They are thus currently administered by frequent injection or infusion, which is less user friendly than the options available for small-molecule drugs and could eventually limit the widespread use of protein therapeutics by physicians and patients [3,4].

Hence, great attention has been paid to a promising drug delivery system, which could protect protein drugs from enzymatic degradation and provide a sustained-release effect. An appropriate delivery carrier, which can meet the above requirements and has been accepted for the delivery of protein drugs, is polymeric microparticles [5,6]. Furthermore, the pulmonary drug delivery system used for biomacromolecular drugs could protect protein drugs from enzymatic degradation because of no first-pass effect. In addition, the thin pulmonary alveoli wall and fast blood circulation for the drug’s absorption and transportation to the whole body are desired for the treatment of systemic diseases such as osteoporosis and weak immunity [7,8,9,10]. At present, the forms of inhalation system based on polymer to deliver the protein and other biomacromolecular drugs include liposomes, nanoparticles, microparticles and porous microparticles (PMs). With a light density (<0.4 g·cm^−3^) and a proper geometric mean diameter (Dg) (10–30 μm) and aerodynamic diameter (Da) (<4.7 μm), PMs with the embedded drugs have been the first choice as inhalers for their excellent aerodynamic performances.

The conventional methods to prepare PMs, such as spray drying (SD), spray freeze drying (SFD) and double-emulsion solvent evaporation all face the same problem of failing in effectively removing the organic solvent to a very low concentration, which is important to ensure safe and stable microsphere products. To meet the requirements for pulmonary drug delivery, a promising method to prepare PMs is of urgent necessity.

Due to its properties of having very low solvent residue, and being environmentally friendly and low cost [11], supercritical CO_2_ (SCCO_2_) has been employed in the field of production of PMs because of the various roles it can play: drying agent [12,13,14,15], gas foaming agent [16,17,18,19] and porogen [20,21]. The compressed antisolvent (PCA) process, which is based on the SCCO_2_ antisolvent process, has been widely used in producing solid drug-loaded superfine polymer particles. However, the shortcomings of PCA process are its tiny size and closed pores or quite low porosity, which are not suitable for pulmonary drug delivery. To overcome these drawbacks and provide a basis for future research on pulmonary drug delivery, we attempted to perform the PCA process in combination with a water-in-oil emulsion process, since the PCA process has been applied to produce PMs [22,23,24,25]. In this study, ammonium bicarbonate (AB), lysozyme (LSZ) and poly-L-lactide (PLLA) were respectively used as a porogen—the drug model and the polymer matrix—aiming at developing a biomacromolecular-loaded dry powder inhaler formulation for potential application in the future treatment of lung diseases.

## 2. Results and Discussion

### 2.1. Analysis of LSZ-Loaded PLLA PMs (LSZ-PLLA PMs): Morphology, Size, Porosity and Tapped Density Investigation

**Figure 1:**
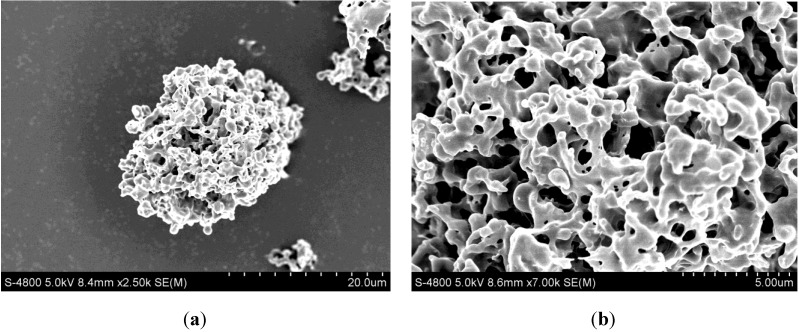
SEM images of lysozyme (LSZ)-loaded poly-L-lactide (PLLA) porous microparticles (PMs) (LSZ-PLLA PMs): (**a**) with magnification of 2.50 k; (**b**) with magnification of 7.00 k.

**Figure 2:**
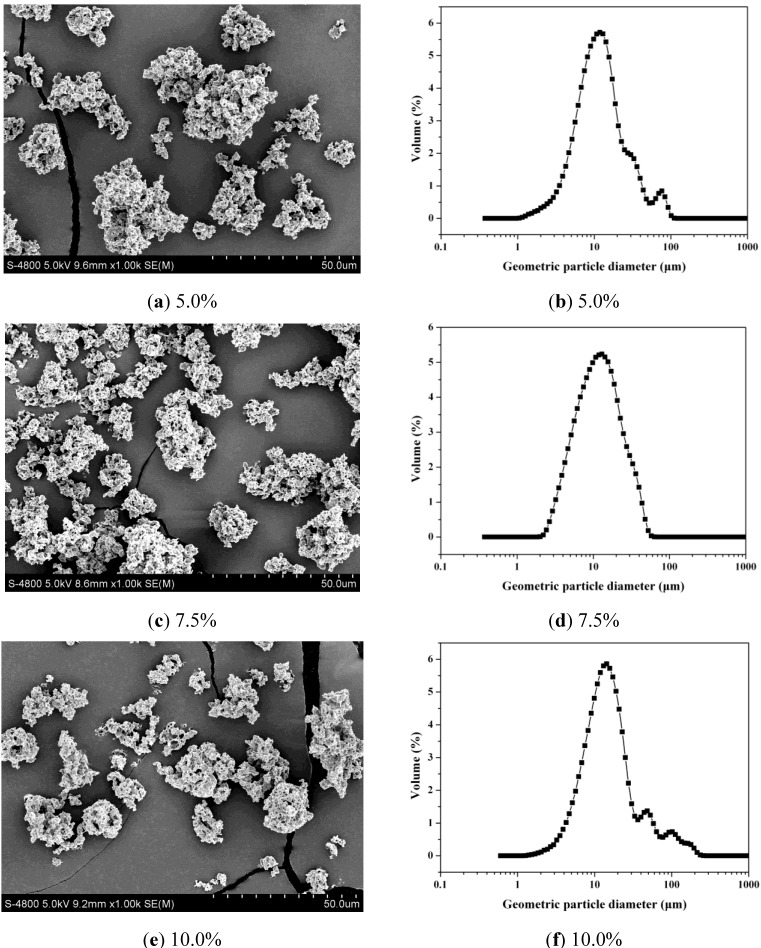
SEM images of LSZ-PLLA PMs of different drug loads (DLs) and their geometric particle size distributions.

The formation of LSZ-PLLA PMs prepared by PCA process was based on antisolvent effect between SCCO_2_ and emulsion. As presented in the process conducted before [26], at the desired pressure and temperature, the emulsion droplets were delivered into autoclave to contact the SCCO_2_, leading to a strong mass transfer between them. Then, dichloromethane in emulsion was expanded and extracted by SCCO_2_; as a result, PLLA microparticles containing AB and LSZ microdroplets were formulated. Afterwards, vacuum drying was applied to remove the AB in microdroplets to produce the LSZ-PLLA PMs. From Figure 1, it is observed that the LSZ-PLLA PMs exhibit a porous morphology and are composed of small particles and fibers, and there are many pores with different sizes, which were generated by the removal of AB and CO_2_. As shown in Figure 2 and Table 1, the harvested LSZ-PLLA PMs have a light density (less than 0.1 g·cm^−3^), and proper Dg (16.9–18.8 μm) and Da (2.6–2.8 μm) meeting the requirements for pulmonary drug delivery. The porosity of LSZ-PLLA PMs decreased from 86.3% to 78.2% with an increase in theoretical drug load (DL) (from 5.0% to 10.0%), indicating that a higher loading of LSZ resulted in a less encapsulation of water phase. The recovery rate of this method to produce LSZ-PLLA PMs was 57.46%, which was obtained by that the mass of products harvested divided by the total mass of the materials (PLLA and Pluronic-F127 (PF-127)).

**Table 1:** Results of aerodynamic properties of LSZ-PLLA PMs.

Sample	Dg [μm]	Da [μm]	FPF [%]	Porosity [%]	Density [g·cm^−3^]	Aperture [μm]
LSZ-5.0%	18.8 ± 2.4	2.6 ± 0.2	66.8 ± 1.5	86.3	0.09 ± 0.02	0.70
LSZ-7.5%	17.5 ± 0.7	2.7 ± 0.4	63.9 ± 1.4	81.2	0.07 ± 0.02	0.79
LSZ-10.0%	16.9 ± 1.9	2.8 ± 0.6	59.2 ± 1.9	78.2	0.10 ± 0.03	0.82

According to our previous study [26], a proper pressure (8 MPa) and temperature (30 °C) were the premise for the formation of micro-scale PLLA particles with excellent pore properties. Within the range of parameters studied, CO_2_ flow rate was the most predominant factor affecting the microparticles’ Dg, which became bigger when the CO_2_ venting rate decreased, since a lower venting speed of SCCO_2_ should maintain the original shape of emulsion, while in the case of higher flow rate, the emulsion would be distorted into small particles with irregular shapes and a non-porous morphology. The water-oil ratio (W/O ratio) was the key parameter affecting their Das, which tended to become smaller when the W/O ratio increased. Oil phase concentration and the emulsion flow rate influenced the viscosity and the size of the initial emulsion droplet, respectively, showing no significant effect on the particle sizes.

### 2.2. Investigation of DL, Encapsulation Efficiency (EE) and *in vitro* Drug Release Profiles

**Figure 3:**
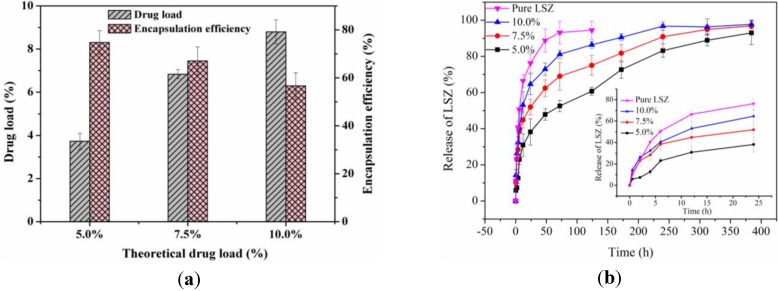
(**a**) DL and EE of LSZ-PLLA PMs; (**b**)* in vitro* release curves.

As shown in Figure 3a, the DL of LSZ-PLLA PMs increased with an increase in theoretical DL, while the EE decreased (74.7%, 67.0% and 56.6%, respectively). This phenomenon, which was similar to the previous study [27], could be explained by the presence of AB leading to the difficulty in encapsulating more LSZ and LSZ, which may precipitate at the interface between the oil phase and the water phase. Figure 3b shows the *in vitro* drug release profiles of LSZ from LSZ-PLLA PMs. The cumulative releases of LSZ-PLLA PMs with different theoretical DLs of 5.0%, 7.5% and 10.0% within 0.5 h were 5.9%, 10.2%, and 14.2%, respectively, which attributed to the release of drug absorbed on the surface of PMs. Since LSZ is of high molecular weight, the release of pure LSZ was almost complete (93.2%) in 72 h; while until 300 h, the release of LSZ from the LSZ-PLLA PMs with different theoretical DLs of 5.0%, 7.5% and 10.0% were 83.2%, 90.9% and 96.7%, respectively, which indicates that the LSZ-PLLA PMs could provide a sustained-release effect.

### 2.3. Characterization of Physicochemical Properties of LSZ-PLLA PMs

**Figure 4:**
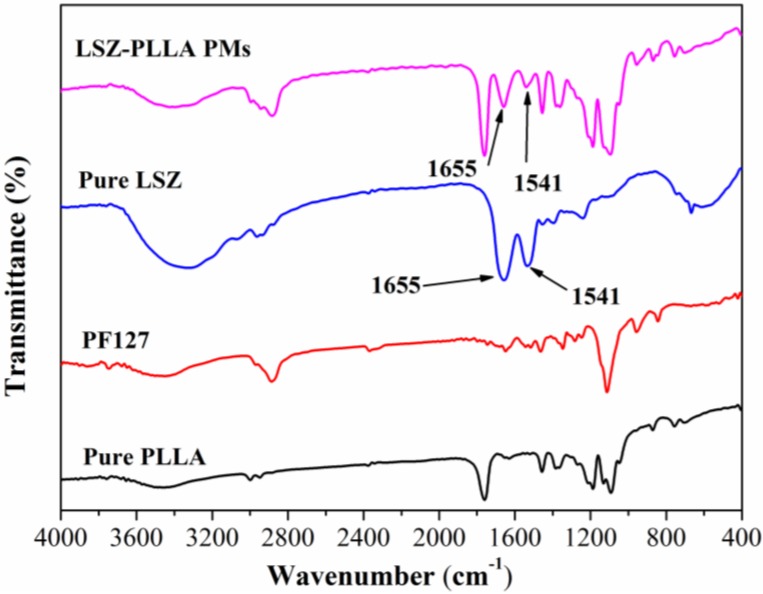
Fourier transform infrared (FTIR) spectra of pure LSZ, pure PLLA, PF127 and LSZ-PLLA PMs.

To investigate the changes in molecular structures of protein before and after the PCA process, Fourier transform infrared (FTIR) analysis of the LSZ-PLLA PMs, pure LSZ, PF-127 and PLLA were carried out. As shown in Figure 4, the characteristic peaks of α-helix in LSZ (1655 cm^−1^) [28] could be found in both pure LSZ and LSZ-PLLA PMs, as well as the peaks of C–N stretching vibration and N–H bending vibration at 1541 cm^−1^ in molecular skeleton of LSZ. The results show that the LSZ was successfully loaded in the PLLA PMs, and almost no changes occurred in the molecular structures of LSZ after PCA process, which reveals that the method presented here would be useful in the application of a drug delivery system for protein-polymers.

**Figure 5:**
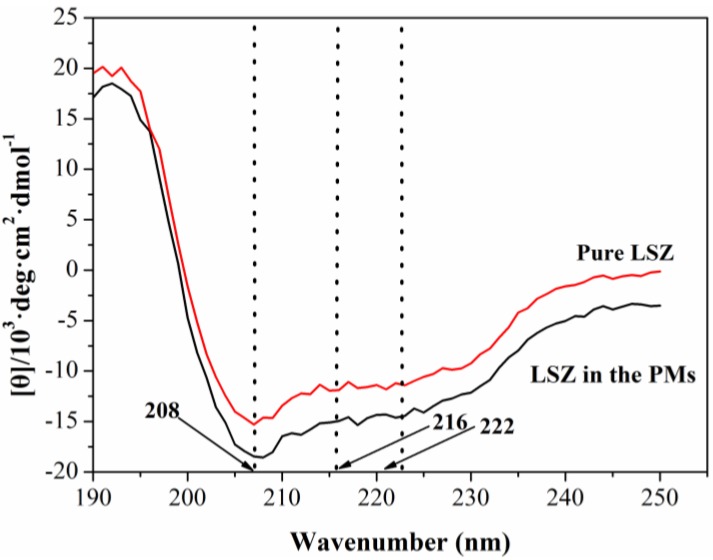
Circular dichroism (CD) spectra of pure LSZ and LSZ from LSZ-PLLA PMs.

To further determine the effect of the PCA process on the secondary structure of LSZ, the circular dichroism (CD) spectra measurements of the LSZ before and after the PCA process were also carried out. As illustrated in Figure 5, the difference between the CD spectroscopy of the pure LSZ and the LSZ loaded in the PLLA PMs were not significant, and close to the wavenumbers 208 nm, 216 nm and 222 nm, the test sample exhibited negative peaks as pure LSZ, indicating that there was almost no change in the α-helix or β-sheet of LSZ after the PCA process.

**Figure 6:**
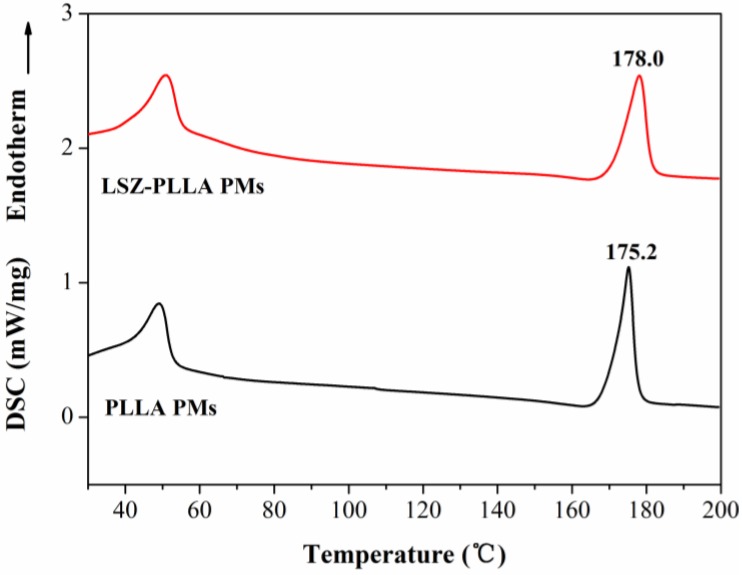
Differential scanning calorimetry (DSC) curves of blank PLLA PMs and LSZ-PLLA PMs.

**Figure 7:**
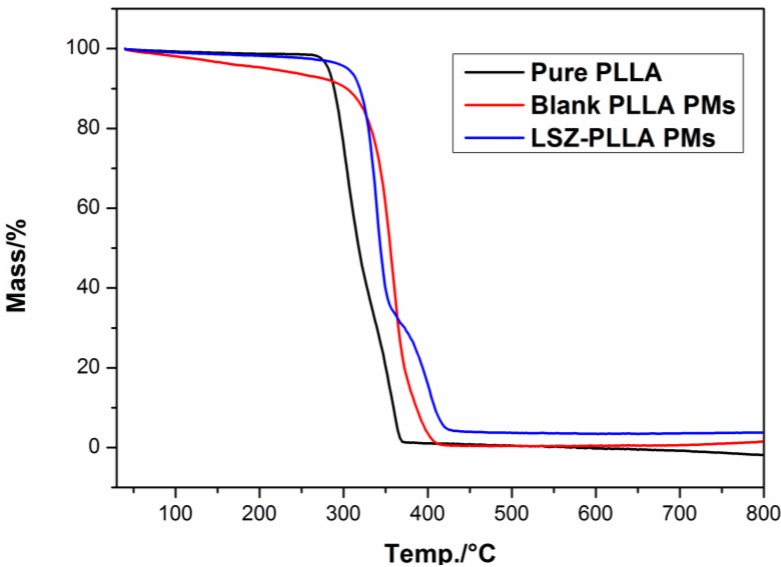
Thermogravimetric analysis (TGA) curves of pure PLLA, blank PLLA PMs and LSZ-PLLA PMs.

As shown in Figure 6, the results of differential scanning calorimetry (DSC) measurement revealed that compared with the blank PLLA PMs, the melting temperature of PLLA in LSZ-PLLA PMs shifted from 175.2 °C to 178.0 °C, exceeding the melting temperature of pure PLLA, which indicated that the thermal stability of PLLA was improved after precipitation with LSZ. A similar phenomenon was observed from the results of thermogravimetric analysis (TGA), which were presented in Figure 7. The possible reason for this may be that hydroxyl of LSZ promoted the degradation of PLLA [29], while the barrier effects and thermal stability of LSZ affected the thermal decomposition of PLLA. So, the thermal stability of PLLA in LSZ-PLLA PMs was improved to some extent. Above all, it is indicated that this emulsion-combined PCA process would be a feasible method in loading macromolecular drugs without altering their properties and ensuring pulmonary drug delivery.

### 2.4. Activity Assay of LSZ before and after Precipitation Process

**Figure 8:**
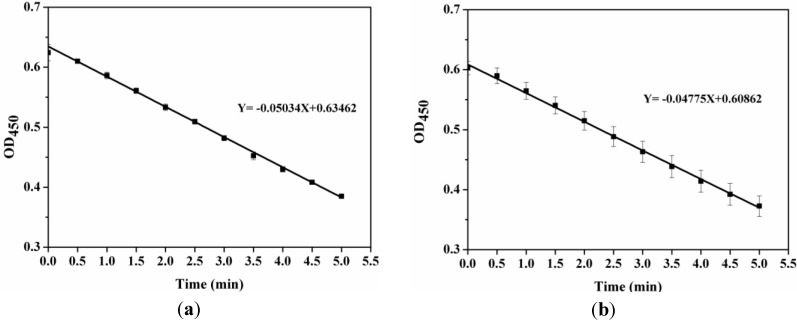
Enzyme activity curves of (**a**) pure LSZ; (**b**) LSZ from LSZ-PLLA PMs.

Figure 8 shows the enzyme activity curves of pure LSZ (*Y* = −0.05034*X* + 0.63462) and LSZ released from the LSZ-PLLA PMs (*Y* = −0.04775*X* + 0.60862). The slope of curve was defined as the change in absorbance over time at 450 nm, which was related to enzyme activity. After the PCA process, the retention rate of enzyme activity of LSZ was 94.85%, illustrating that the process had a minor influence on the senior structure of LSZ and the precipitation with polymer could prevent the inactivation of enzyme. However, as reported in a previous study [30], the activity of LSZ was improved after the process of solution enhanced dispersion by supercritical fluids, which may be explained by the occurrence of a better combination of active groups of LSZ and substrate. The slight loss in specific enzymatic activity of LSZ here might be explained by the low efficacy of emulsifier PF-127 in preventing LSZ unfolding at the water-methylene chloride interface and entrapment of LSZ in the PLLA layer [31].

## 3. Experimental Section

### 3.1. Materials

PLLA (MW 50,000, 1.5 dL/g) was purchased from the Jinan Daigang Co., Ltd. (Jinan, China). PF-127, Micrococcus Lysodeikticus and AB were purchased from Sigma-Aldrich (USA). LSZ (>98% purity) was purchased from Changcheng pharmaceutical Co., Ltd. (Shanghai, China). Dichloromethane (99.8% purity) was purchased from the Sinopharm Chemical Reagent Co., Ltd. (Shanghai, China). CO_2_ of 99.9% purity was supplied by the Rihong Air Products Co., Ltd. (Xiamen, China). All other compounds were of analytical purity.

### 3.2. Methods

#### 3.2.1. Preparation of LSZ-PLLA PMs

In this section, the oil phase was obtained by dissolving the PLLA and emulsifier PF-127 in dichloromethane at a concentration of 2.3%. 200 μL of LSZ aqueous solution with different concentrations was first added into the oil phase, followed by an ultrasonication at 200 W for 30 s. Taking the water-oil ratio as 2.0/20 (v/v), AB aqueous solution of 250 mg·mL^−1^ was added into the prepared emulsion and an ultrasonication was used to ensure that both porogen and LSZ were homogeneously mixed within the oil phase. Afterwards, the finally-prepared emulsion was subjected to the PCA process.

As described in a schematic diagram shown in previous study [26], the apparatus of fabrication process consists of three major components: a CO_2_ supply system, a solution delivery system and an autoclave with a volume of 500 mL. At the beginning of an experiment, the CO_2_ fed from a CO_2_ cylinder was cooled down to around 0 °C by using a cooler to ensure the liquefaction of the gas and also to prevent cavitation, followed by delivery using a high pressure meter pump. Then, a heat exchanger was used to preheat the liquefied CO_2_ to the desired operating temperature before it entered the autoclave, which was incubated in a gas bath to keep the temperature constant during the experiment. When the desired pressure (8 MPa) was reached, a steady flow of CO_2_ (1000 L·h^−1^) was maintained by adjusting the frequency of CO_2_ pump and regulating a downstream valve of CO_2_. Then, the emulsion in the syringe-like cylindrical device was fed into the autoclave through a nozzle (inner diameter 160 μm) at a flow rate of 4.0 mL·min^−1^. When the spraying of emulsion was finished, a washing process of 30 min was performed by continuously pumping fresh CO_2_ into the autoclave was used to remove the residual organic solvent. After washing, the autoclave was slowly depressurized and the powder products containing AB microdroplets could be harvested. After being dried at 50 °C for 1 h, AB was decomposed and the LSZ-PLLA PMs were obtained for further characterizations.

#### 3.2.2. Characterization of Surface Morphology

Scanning electron microscopy (S-4800 UHR FE-SEM, Hitachi, Japan) was applied to investigate the surface morphology of the LSZ-PLLA PMs. The samples were absorbed onto the conducting resin and then sprayed with gold under vacuum conditions.

#### 3.2.3. Investigation of Particle Size, Porosity and Tapped Density

Laser particle size analyzer (LS13-320, Beckman Coulter, USA) was applied to measure the geometric particle sizes. Da is a key parameter which determines whether the PMs could be effectively delivered into the deep lung. It has been widely accepted that an eight-stage Andersen Mark II cascade impactor (ACI 20-810, Thermo Scientific, USA) was used to sort and collect the powder according to the Da cutoffs. The capsule, containing 10 mg of the sample, was placed inside the dry powder inhaler device and then the powder released from a hole. A tube with a 90° bend, connecting the inhaler device and ACI, was employed to act as a simulated throat. When operated at a flow rate of 28.3 L·min^−1^, the powder was absorbed into the ACI, passing through each stage of different Da cutoffs, leading to some of it being deposited at each stage. When each operation was finished, the weight of powder at each stage was recorded for the calculation of the Da and the FPF, as a Da less than 4.7 μm was an important index for aerodynamic performance of PMs. The measurement for a sample was conducted in triplicate. The aperture and porosity of the samples were also measured by using a high-pressure mercury intrusion porosimeter (Pore Master GT 60, Quantachrome Instruments, Boynton Beach, FL, USA). The tapped density of the samples was determined by a tapping apparatus (GH-01, Matsuhaku Electronic Co., Ltd., Taiwan, China).

#### 3.2.4. Measurement of DL and EE

DL was defined as the total content of the drug entrapped in the PMs, and the EE was described as percentage of the drug encapsulated in polymer matrix of the PMs. For the determination of the DL, 10 mg of LSZ-PLLA PMs was dissolved in dichloromethane, followed by adding the phosphate-buffered solution (PBS, pH 7.4). Then, magnetic stirring and gentle heating was used to evaporate dichloromethane. Finally, the solution contained LSZ was filtered with 0.22 μm membrane and the concentration of LSZ was analyzed by a UV spectrophotometer (Unic UV-2800, Yongxian Electronic Instrument Co., Ltd., Hong Kong) at 280 nm. According to Equation (1) listed below, the DL could be calculated. For determination of the EE, a sample of 10 mg was dispersed in PBS to wash off the unencapsulated LSZ. Then, the resulting suspension was filtered with a 0.22 μm membrane, and the amount of LSZ on the surface was determined. The content of the encapsulated protein could be confirmed according to Equation (2) by subtraction between the total amount of LSZ and the amount of LSZ on the surface.

DL = *W_1_*/*W_2_* × 100% (1)

EE = *W_3_*/*W_1_* × 100% (2)

where *W_1_*, *W_2_* and *W_3_* represent the total weight of LSZ in LSZ-PLLA PMs, the weight of LSZ-PLLA PMs and the weight of the protein encapsulated, respectively. Each experiment was done in triplicate.

#### 3.2.5. Study of Drug Release Profiles

Taking 10 mg pure LSZ as the contrast, LSZ-PLLA PMs of different drug loading were placed in dialysis bags, which were placed in a centrifuge tube containing 30 mL PBS, followed by incubating in a shaking water bath at 37 °C and 60 rpm. At a predefined time point, 5 mL of solution outside the bag was abstracted to measure the concentration of LSZ in terms of the accumulative release percentage of LSZ (wt/wt, %). Meanwhile, 5 mL of fresh PBS was added to maintain the total volume of solution for the next measurement. Each experiment was conducted in triplicate.

#### 3.2.6. Characterization of Physicochemical Properties of LSZ-PLLA PMs

FITR was conducted to estimate the influence of process on chemical composition of the LSZ on an FITR 8400S (SHIMADZU). For FITR analysis, the samples were gently mixed with KBr. DSC and TGA were used for evaluation of thermodynamics properties of the materials before and after the precipitation process, in which the samples were heated from 30 °C to 200 °C and 40 °C to 800 °C at a rate of 10 °C·min^−1^, respectively. CD spectroscopy was applied to investigate the change of secondary structure of LSZ after PCA process. Before the test, the sample was suspended in ultrapure water, and then LSZ solution was obtained by filtering the suspension. The measurement was conducted in the scanning wavenumber of 190–250 nm, a scanning speed of 100 nm·min^−1^, a flow rate of N_2_ of 5–10 L·min^−1^ and sensitivity for analyses of 5 mdeg·cm^−1^.

#### 3.2.7. Determination of Enzyme Activity Retention Rate of LSZ

The retention rate of LSZ activity after treatment was measured by spectrophotometry. LSZ-PLLA PMs were suspended in the PBS to form a suspension, which was filtered with 0.22 μm membrane to obtain the solution of LSZ. 10 μL of the harvested solution was added to 2 mL of Micrococcus Lysodeikticus suspension. The absorbance value of bacterial suspension was investigated using a UV spectrophotometer (Unic UV-2800, Yongxian Electronic Instrument Co., Ltd., Hong Kong) at 450 nm at every 30 s, which lasted for 5 min. The retention rate of LSZ activity was calculated as the ratio of specific enzyme activity of LSZ released from LSZ-PLLA PMs to that of the pure LSZ.

## 4. Conclusions

The LSZ-PLLA PMs with a light density and a proper Dg and Da, which met the requirements for pulmonary drug delivery, were successfully prepared using a compressed CO_2_ antisolvent process, without any changes in chemical composition and secondary structure of LSZ. The loading of LSZ within the porous PLLA matrix not only preserved the activity of LSZ but also increased the thermal stability of PLLA, which is favorable for further application. The LSZ-PLLA PMs possessed a sustained-release efficacy with relatively high DL and EE. This study indicates that the emulsion-combined PCA process could be a feasible and promising method in producing biomacromolecular drug-loaded PMs for pulmonary drug delivery.
